# Interaction effects between sender and receiver processes in indirect transmission of *Campylobacter jejuni* between broilers

**DOI:** 10.1186/1746-6148-8-123

**Published:** 2012-07-25

**Authors:** Bram AD van Bunnik, Thomas J Hagenaars, Nico M Bolder, Gonnie Nodelijk, Mart CM de Jong

**Affiliations:** 1 Central Veterinary Institute of Wageningen UR, P.O. Box 65, Lelystad, AB, 8200, The Netherlands; 2 Quantitative Veterinary Epidemiology, Department of Animal Science, Wageningen University, Wageningen, The Netherlands

## Abstract

**Background:**

Infectious diseases in plants, animals and humans are often transmitted indirectly between hosts (or between groups of hosts), i.e. via some route through the environment instead of via direct contacts between these hosts. Here we study indirect transmission experimentally, using transmission of *Campylobacter jejuni (C. jejuni)* between spatially separated broilers as a model system. We distinguish three stages in the process of indirect transmission; (1) an infectious “sender” excretes the agent, after which (2) the agent is transported via some route to a susceptible “receiver”, and subsequently (3) the receiver becomes colonised by the agent. The role of the sender and receiver side (stage 1 and stage 3) was studied here by using acidification of the drinking water as a modulation mechanism.

**Results:**

In the experiment one control group and three treatment groups were monitored for the presence of *C. jejuni* by taking daily cloacal swabs. The three treatments consisted of acidification of the drinking water of the inoculated animals (the senders), acidification of the drinking water of the susceptible animals (the receivers) or acidification of the drinking water of both inoculated and susceptible animals. In the control group 12 animals got colonised out of a possible 40, in each treatment groups 3 animals out of a possible 40 were found colonised with *C. jejuni.*

**Conclusions:**

The results of the experiments show a significant decrease in transmission rate (β) between the control groups and treatment groups (p < 0.01 for all groups) but not between different treatments; there is a significant negative interaction effect when both the sender and the receiver group receive acidified drinking water (p = 0.01). This negative interaction effect could be due to selection of bacteria already at the sender side thereby diminishing the effect of acidification at the receiver side.

## Background

Many infectious diseases, both plant related and animal related (including human diseases) spread via indirect transmission instead of direct transmission. For many plant diseases this process is well understood in terms of fungal spores travelling from one host to the next [1,2]. However for animal diseases indirect transmission is not well understood. For a number of these diseases we have some information on the routes of indirect transmission. For example, in the context of between-farm transmission of infection, indirect pathways such as sharing of equipment and between-farm movement of vehicles and humans are reported as possible routes of transmission [3-7]. Also for a number of human infections (for example hospital infections such as MRSA) indirect transmission has been implicated. Typically there is a lack of insight into the detailed mechanisms underlying indirect transmission.

More insight would help to develop better prevention measures against this form of transmission.

In a simple tentative representation the process of indirect transmission can be thought of as consisting of three stages. As a first stage there is an infectious host (the sender) that excretes an agent in the environment. During stage two, the agent has to travel through the environment (via some route or multiple routes) to the susceptible host (the receiver) that can become infected or colonised by the agent in stage three. Using this representation in stages as a reference frame helps us to study how these sub-processes connect and, possibly, interact with each other, thus improving our understanding of the mechanisms of indirect transmission.

In this study we consider only stage 1 and 3 of our representation of indirect transmission. For this study an indirect transmission experiment was carried out. As a model system for indirect transmission we used the spread of *Campylobacter jejuni* (*C. jejuni*) between spatially separated broiler chickens. For colonisation with *C. jejuni* the faecal-oral route is the most likely route of transmission. The faecal-oral route consists mainly of indirect transmission, making this system a suitable model system for studying indirect transmission. Furthermore, we know from previous studies that the rate of indirect transmission can be decreased by acidification of the drinking water [8-10]. Here we used this intervention to obtain more insight into the different stages of indirect transmission and their possible interaction. In the experiment we used a novel setup consisting of three treatment groups, one group in which the (infectious) sender animals received acidified drinking water, one group in which the (susceptible) receiving animals received acidified drinking water and one group in which both sender and receiving animals were given acidified drinking water. From the experimental observations the per day chance of colonisation, the effect of acidification of the drinking water, both at the sender and at the receiver stage, and possible interaction effects between acidification of the sender stage and the receiver stage were estimated.

## Methods

### Experimental design

**Figure 1:**
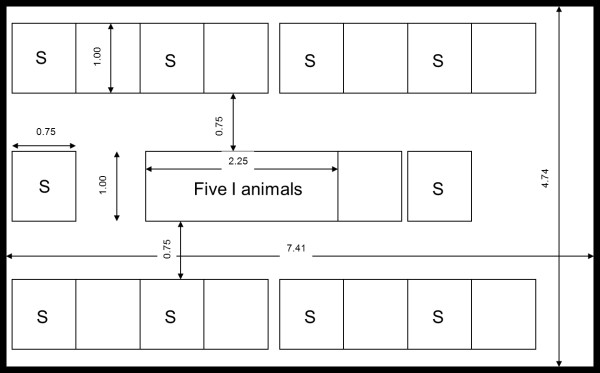
**Schematic overview of the housing of the experimental groups of five infectious sender animals (denoted with I) in the centre cage and ten susceptible receiver animals (denoted with S) in the cages surrounding this centre cage.** Alongside the arrows distances are given in meters.

Each experiment consisted of one control group and three treatment groups. The experiment was replicated four times. In each group, five chicks were orally inoculated with *C. jejuni* by gavage. The five inoculated chicks (sender animals) were housed together in one cage in the centre of an experimental room (a climate controlled room in an experimental facility). Ten chicks (receiver animals) were housed individually in cages surrounding this centre cage placed at a minimum distance of 75 cm (see Figure 1) and exposed indirectly to the inoculated sender animals.

The three different treatments were as follows:

1) Acidification of the drinking water of the susceptible animals (indicated as S+);

2) Acidification of the drinking water of the inoculated animals (indicated as I+);

3) Acidification of the drinking water of both inoculated animals and susceptible animals (indicated as S+ I+).

To measure indirect transmission, all source and recipient animals were sampled daily by means of a cloacae swab (see section on Sampling). These swabs were tested within two hours after sampling in the laboratory for the presence of *C. jejuni*. If a tested recipient animal was found *C. jejuni* positive, the animal was considered colonised and was immediately removed from the experiment to avoid having to deal in the analysis with multiple cages contributing to the infection pressure. The removed animals were euthanized and cecum was removed for further investigation for the presence of *C. jejuni.*

The experiment ended 35 days post inoculation. All remaining sender and receiver animals (that had not been found positive until that moment) were euthanized and cecum was removed and further investigated for the presence of *C. jejuni.* All animal experiments were in compliance with national and institutional regulations and as such approved by the institute's ethical committee.

### Housing

One-day old broilers (type Ross 308) were obtained from a commercial hatchery. At day 7 and day 12 after arrival, cloacal swabs taken from each chick confirmed the absence of *C. jejuni*. For each of the four experiments from the day of arrival (day 0) until 12 days post-arrival, 60 chicks were housed together in one experimental room, divided in two groups of 30 animals. One group received tap water, the other acidified drinking water. On day 12, the control groups and the treatment groups were formed from the two groups, i.e. for the S+ group 10 animals were randomly taken from the acidified drinking water group and 5 animals from the tap water group; for the I+ group 10 animals were randomly picked from the tap water group and 5 from the acidified drinking water group; for the S+ I+ group 15 animals were taken from the acidified drinking water group; and finally for the control group 15 animals were taken from the tap water group. Each treatment group and the control group was placed in its own experimental room, five chicks (sender animals) housed together in one centre cage and ten chicks (receiver animals) individually housed in ten cages surrounding the centre cage as shown in Figure 1. The cages were placed directly on the floor.

All chicks were housed on wood shavings and the drinking water was supplied through a nipple drinking system. In each set-up, the drinking nipples in the cages on the long sides of the area were supplied from one common water container each, while the centre cage and the two cages along the short side each had a separate drinking water supply. This precluded transmission via a shared drinking water system.

### Inoculation

For inoculation, the *C. jejuni* strain 356 [11] was used. The strain was freshly cultured in hearth infusion broth (microaerobically, 37°C, overnight) and diluted in buffered peptone water to obtain the intended inoculation dose (± 1*10^6^ CFU/ml). The precise concentration (CFU/ml) of *C. jejuni* in the administered inoculum was determined by plating on modified cephoperazone charcoal deoxycholate agar (mCCDA) (Oxoid CM 793) with selective supplement (Oxoid CM 155) before and after the inoculation of the animals. Sender animals were inoculated 14 days after arrival with 1 ml inoculum. All animals were tested positive for Campylobacter within 2 days after inoculation.

### Treatment

For the acidification of the drinking water a commercial acid (Forticoat®, Selko BV) was diluted until a final pH of 4 (approximately 2 ml acid on 1 litre water). Active ingredients of the commercial acid are: sorbic acid, formic acid, acetic acid, lactic acid, propionic acid, ammonium formate, L-ascorbic acid, citric acid, mono- and diglycerides of edible fatty acids and 1,2–propanediol.

### Sampling and testing

To measure indirect transmission, all animals were tested by means of a cloacae swab. After an inoculated chick (sender animal) was found positive for *C. jejuni* on three consecutive days, swabs for those chicks were taken weekly instead of daily. For the susceptible chicks (receiver animals) swabs were taken once a day throughout the experiment. On days when both inoculated and susceptible animals were to be sampled in each group, the susceptible animals were sampled first. Animals were sampled every day in a fixed order. If a receiver animal tested positive for *C. jejuni,* the animal was immediately removed from the experiment and sacrificed for further investigation of the cecum.

Samples were collected using sterile swabs (sterile plain dry swabs, Copan Diagnostics Inc., USA). Swabs were directly plated on mCCDA, incubated microaerobically at 41.5°C for 48 hours and examined for the presence of *C. jejuni*. The swab was then placed in Preston enrichment medium (Nutrient Broth no. 2, Oxoid CM0067 with Campylobacter selective supplement (Oxiod SR0204E) and Campylobacter growth supplement (Oxoid SR0232E)) and incubated microaerobically at 41.5°C for 24 hours. After incubation, it was plated on mCCDA and incubated microaerobically at 41.5°C and examined for the presence of *C. jejuni* after 24 and 48 hours.

### Hygienic measures

Before the start of the experiment, all experimental rooms were cleaned and disinfected with formaldehyde. Subsequently, samples were taken from 12 different areas inside the room to check for the absence of *C. jejuni*.

To prevent animal caretakers from acting as a vector of transmission, during the entire experiment strict hygienic measures were used. Clean overalls were used at every entry into the experimental rooms. A pair of boots was dedicated to each room, cleaned on entering and exiting it by means of wading through a chlorinated bath (Suma Tab D4, JohnsonDiversity). Sterile gloves were changed between handling individual animals.

### Quantification of transmission

Differences in total number of infected animals were tested using a Fisher Exact test. To quantify the transmission between sender and receiver animals a stochastic susceptible-infectious (SI) type model [12] was used. This model can be written in terms of state changes; i.e. if a susceptible receiver animal in the experiment becomes colonised, and is subsequently removed when found positive, we can denote this as S→ \S-1. The rate of this state change is βSI, with a different β for each treatment. From the experimental observations the parameter β was estimated for the different treatments as in [13]. In addition, an analysis of the interaction, if any, between acidification of the sender side or the receiver side was carried out. This latter analysis uses a multiplicative model (additive on log-scale) for the effect of treatments and their possible interaction. Estimation of β was carried out by means of a GLM [14]. To this end the data from all repetitions were pooled and represented in the form of (S(t), C(t), Δt), where S(t) is the number of susceptible receiver animals at the beginning of a time period with length Δt, C(t) is the number of new colonisations that occurred in the time period (t, t + Δt). In our model the number of new cases is binomially distributed:

(1) $$C\left(t,t+\text{Δ}t\right)\sim Bin\left(S\left(t\right),p_{\text{inf}}\left(t,t+\text{Δ}t\right)\right)\text{,}$$

with parameter ${\text{p}}_{\text{inf}}\left(t,t+\text{Δ}t\right)=1-\exp\left(-\beta \;I_{\mathrm{treatment}}\;\text{Δ}t\right)$and binomial totals S(t).

This can be rewritten as a GLM with a complementary log-log link function and log(I_treatment_ Δt) as the offset variable [14-16]. We note that because the number of infectious animals is constant over time and new colonisations are removed upon detection, in this setup the estimate for the transmission parameter β is equivalent to the force of infection (β·I_0_).

## Results

**Figure 2:**
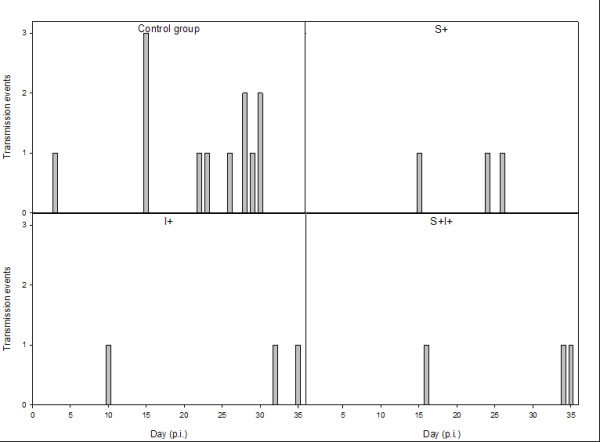
**Experimental results showing the number of new infections per treatment group per day after inoculation.** S+ indicates acidification of the drinking water of the susceptible side. I+ indicates acidification of the drinking water of the infectious side and S+ I+ indicates acidification of the drinking water of both susceptible and infectious animals, p.i. = post inoculation.

Table 1 shows the number of colonised animals per treatment group per repetition of the experiment and the total number of colonised animals per treatment. The control group received tap water, while the treatment groups received acidified drinking water at either the sender side, the receiver side or both. In total we observed twelve transmission events in the control group and three transmission events in each treatment group. One susceptible animal died in the control group. Analysis of these overall data shows a significant reduction in transmission between inoculated sender animals and exposed receiver animals for the treatment groups compared with the control group (p < 0.01 for all groups, Fisher Exact Test). No significant differences in transmission were found between the three treatment groups. We found no correlation between the spatial order of colonisation of recipient animals and the order of sampling of the animals. Figure 2 shows the distribution of transmission events in time. For all groups the transmission parameter β was calculated by GLM from these data. The results are shown in Table 2. For the control group the probability per day of infection (β) was found to be 0.00175 day^-1^ and for each treatment groups 0.00044 day^-1^. 

**Table 1:** Number of positive broilers per experiment repetition and total number of exposed animals per treatment group

**Treatment**	**Repetition**				**Total positive**	**Total exposed**
	**1**	**2**	**3**	**4**		
**Control**	9	2	1	0	12	39^†^
**S+**	1	1	0	1	3	40
**I+**	1	0	0	2	3	40
**S+ I+**	1	2	0	0	3	40

^†^: One animal died during the experiment.

S+ indicates acidification of the drinking water of the susceptible side. I+ indicates acidification of the drinking water of the infectious side and S+ I+ indicates acidification of the drinking water of both susceptible and infectious animals.

**Table 2:** Estimation of the per day chance of infection for different treatment groups

**Treatment**	**Estimate of β (CI)**
**Control**	0.00175 (0.00129 - 0.00239)
**S+**	0.00044 (0.00023 - 0.00085)
**I+**	0.00044 (0.00023 - 0.00087)
**S+ I+**	0.00044 (0.00022 - 0.00085)

S+ indicates acidification of the drinking water of the susceptible side. I+ indicates acidification of the drinking water of the infectious side and S+ I+ indicates acidification of the drinking water of both susceptible and infectious animals. CI = 95% confidence interval.

No significant difference was found between the three treatments. This indicates that when one side is acidified there is no additional effect of acidification at the other side. This finding is confirmed by analysing the data as a multiplicative model, which yields a significant negative interaction effect. The results of this test are given in Table 3. A negative interaction effect means that acidifying the drinking water of both sides has less effect than the multiplication (addition on a log-scale) of the two one-side acidification effects. The small difference in the Akaikes Information Criterion (AIC) for the univariate model (AIC = 186.31) and the model with interactions (AIC = 186.59) suggests that, although the interaction effect is significant, it does not improve the model fit and thus interaction is not necessary to explain the data [17]. 

**Table 3:** Interaction effects between receiver and sender treatment

**Group**	**Estimate**	**Std. error**	**p**
**Control**	−6.346	0.155	<.001
**S+**	−1.368	0.333	<.001
**I+**	−1.388	0.333	<.001
**S+ I+**	1.362	0.534	0.011

S+ indicates acidification of the drinking water of the susceptible side. I+ indicates acidification of the drinking water of the infectious side and S+ I+ indicates acidification of the drinking water of both susceptible and infectious animals. Estimates given are for the natural logarithm of multiplicative effects on the transmission parameter.

## Discussion

The role of the sender and receiver was studied here by using indirect transmission of *C. jejuni* between spatially separated broilers as a model system with acidification of the drinking water as a modulation factor.

The results of this experiment show that acidification of the drinking water significantly reduced the transmission of *C. jejuni* between spatially separated animals. This finding is in line with earlier studies [8-10,18]. Furthermore we found that acidification of either the drinking water of sender animals or that of receiver animals or both is not significantly different. Moreover, we do find a significant negative interaction effect between acidification on the sender and on the receiver side. This indicates that the effect of acidification of the drinking water of both sender and receiver animals is not a multiplicative effect. A possible explanation arises from hypothesizing selection of agent by acidification. When both inoculated and susceptible are acidified it is plausible that agent selection takes place at the inoculated (sender) side. Only agents capable of surviving an acidified environment (either inside or outside the host) will be able to get to the lower tracts of the intestine of the host and reproduce. Some evidence exists that *C. jejuni* has a mechanism of surviving in a stressful environment. For *C. jejuni* is known that the bacteria can go in a “dormant” state, called the viable but non-culturable state (VBNC) [19]. It has also been reported that these VBNC bacteria are able to return to a culturable state and cause an infection or colonisation [20]. When these (selected) agents are then secreted and transported to the susceptible animals (receivers) the acidified drinking water on this side might have less or no effect; resulting in the same transmission rate as found from acidification of either the sender or the receiver side.

The negative interaction effect indicates that it may be too simple to model indirect transmission probabilities as a product of probabilities of sub-processes. In particular the way in which the effect of intervention measures are represented in (mathematical) models needs to be considered carefully. Most between-farm transmission models do not consider the possibility of an interaction between different measures against (indirect) transmission [21,22]; instead transmission is modelled as a product of (decreased) probabilities. If there is indeed an interaction effect this may lead to an overestimation of the effect of interventions. This is dependent on whether the intervention causes a selection pressure on the pathogen, and whether the selection is fast enough to occur before the (selected) agent reaches new susceptibles (other farms); in those circumstances a control measure could have less effect than previously estimated. A recent and important example of this is the antibiotic resistance in bacteria.

As mentioned before the acidification of either drinking water or feed has been found to reduce pathogen transmission before in different studies. Therefore the results of this study are relevant too for other host-agent systems, in particular those where the faecal-route is the most important route of transmission. Van Gerwe et al. estimated a transmission parameter (β) for direct Campylobacter transmission of 1.04 day^-1^[23]. Comparing this with our estimate of 0.002 day^-1^ for indirect transmission, it is clear that indirect transmission is a less efficient process than direct transmission. This does not mean however that indirect transmission is less important epidemiologically. In fact, the spread of *C. jejuni* in the poultry industry is most probably a combination of indirect transmission for between-flock spread and direct transmission for within flock spread. The estimates imply that the probability of introduction via indirect transmission into a susceptible flock is generally relatively low (i.e. there can be some delay in time before introduction occurs), once introduced however, Campylobacter may typically spread very fast throughout a flock.

We observed a large variation in the number of colonised broilers between repetitions for the control group, as is shown in Table 1. There are three repetitions with a relatively low number of infections (repetitions 2, 3 & 4) and one repetition with a high number of infections (repetition 1). We chose, however, to pool the control repetitions for two reasons: first, we have previously found a significant effect of acidification of the drinking water [10], indicating that the repetition 1 is not a rare outlier. Second, unpublished data from four repetitions with normal tap water in a later experiment show two repetitions with the intermediate number of 4 infections, indicating that the current repetition 1 is not a very strong outlier.

To get more detailed insight in the role of sender and receiver in indirect transmission further experiments should be carried out. An interesting aspect is the effect of dosage of the pathogen on the colonization both with and without acidification of the drinking water as this could provide additional information on the nature of the interaction effect.

## Conclusion

In conclusion, we demonstrated that acidification of either the sender or the receiver side of the transmission chain has an effect on the indirect transmission of *C. jejuni* between broilers. We found that acidification of the drinking water has an effect on the transmission rate compared to a control situation with no acidified drinking water. However this effect is not multiplicative; there is no added advantage of acidifying both sides of the transmission chain.

## Authors’ contribution

BvB participated in the design of the study, carried out the experiment, performed the statistical analysis of the data and drafted the manuscript. TH participated in the design of the study and helped to draft the manuscript. NB and GN participated in the design of the study. MdJ conceived the study and participated in its design. All authors read and approved the final manuscript.
